# Treatment of the Paretic Hand with a Robotic Glove Combined with Physiotherapy in a Patient Suffering from Traumatic Tetraparesis: A Case Report

**DOI:** 10.3390/s23073484

**Published:** 2023-03-27

**Authors:** Federica Bressi, Laura Cricenti, Marco Bravi, Fabiana Pannunzio, Francesca Cordella, Martina Lapresa, Sandra Miccinilli, Fabio Santacaterina, Loredana Zollo, Silvia Sterzi, Benedetta Campagnola

**Affiliations:** 1 Physical Medicine and Rehabilitation Unit, Campus Bio-Medico University Polyclinic Foundation of Rome, 00128 Rome, Italy; 2 Department of Movement, Human and Health Sciences, University of Rome “Foro Italico”, 00135 Rome, Italy; 3 Unit of Advanced Robotics and Human-Centred Technologies, Campus Bio-Medico University of Rome, 00128 Rome, Italy

**Keywords:** spinal cord injury, robotic rehabilitation, hand rehabilitation, upper limb, cervical lesion, quadriplegia

## Abstract

Background: cervical spinal cord injury leads to loss of upper limb functionality, which causes a decrease in autonomy to perform activities of daily living. The use of robotic technologies in rehabilitation could contribute to improving upper limb functionality and treatment quality. This case report aims to describe the potential of robotic hand treatment with Gloreha Sinfonia, in combination with conventional rehabilitation, in a tetraparetic patient. Material: fifteen rehabilitative sessions were performed. Evaluations were conducted pre-treatment (T0), post-treatment (T1), and at two-months follow-up (T2) based on: the upper-limb range of motion and force assessment, the FMA-UE, the 9-Hole Peg Test (9HPT), and the DASH questionnaire. A virtual reality game-based rating system was used to evaluate the force control and modulation ability. Results: the patient reported greater ability to use hands with less compensation at T1 and T2 assessments. Improvements in clinical scales were reported in both hands at T1, however, at T2 only did the dominant hand show further improvement. Improved grip strength control and modulation ability were reported for T1. However a worsening was found in both hands at T2, significant only for the non-dominant hand. The maximum force exerted increased from T0 to T2 in both hands. Conclusion: hand treatment combining physical therapy and Gloreha Sinfonia seems to have benefits in functionality and dexterity in tetraparetic patient in the short term. Further studies are needed to confirm these findings, to verify long-term results, and to identify the most appropriate modalities of robotic rehabilitation.

## 1. Introduction

Spinal cord injury (SCI) is defined as mechanical injury to the spinal cord [1,2], with an annual incidence that ranges between 707,000 and 1,156,000 cases per year in the world [3], while in Italy, it is between 2000 and 2500 cases per year [4].

SCIs have the greatest impact on the bio-psycho-social condition of the person. In fact, the loss of neuromotor skills, sensitivity, and vegetative functions cause the loss or decrease in autonomy in performing activities of daily living (ADLs), influencing the autonomy in everyday life in the psychological, socio-economic, and work aspects, as well as the social–health costs related to the pathological condition itself, with a greater degree of disability the greater the lesion [1,5,6].

In cervical lesions, the disability of the upper limbs assumes particular importance. According to the International Classification of Functioning, Disability and Health (ICF), the recovery of the functionality of the upper limb is essential to promote the autonomy of the patient, since impairments at the body structure and functional level can influence activity limitations and participation restrictions [7,8].

Especially in incomplete injuries, which allow a margin of functional recovery, it is essential that rehabilitation focuses on this aspect, in order to make the patient autonomous in his life, limiting the level of assistance by the caregiver and, consequently, making the patient as independent as possible.

To date, conventional physiotherapy (PT), together with occupational therapy (OT), are considered the main approaches used in patients with SCI [9]. However, in recent years, there has been an increasing application of robotic devices and virtual reality (VR) in the rehabilitation program of these patients. These tools provide additional opportunities compared to traditional treatment: they allow the administration of more specific, repetitive, and high-intensity tasks, which also stimulate the patient’s cognitive aspect and enhance neuronal neuroplasticity thanks to interactive and engaging exercises [10,11].

Morone et al. [5] highlighted how robotic therapy is feasible and safe for patients affected by cervical SCI: the results from this work pointed out an initial positive effect of robotic therapy on arm functionality and quality of movement in addition to conventional therapy. Additionally, De Miguel-Rubio et al. [6], who analyzed the combination of virtual reality and robotics for treatment of patients with SCI, showed an increase in residual shoulder mobility [12] and a significant improvement in ADL [13] due to the improvement of hand and upper limb functionality.

Although the relevant findings of the previous works, studies in SCI patients were relatively few: these results are preliminary, and both reviews agreed on the need for further studies to confirm the obtained results.

Among the robots used for the upper limb, the Gloreha Sinfonia (Idrogenet S.R.L., Brescia, Italy) could be useful for verifying the results achieved and expanding the functionality of the hand, which is a difficult area to recover, as it allows executing different, complex, and fine movements. The Gloreha, with its different modalities of movement assistance of the fingers and of the hand, can facilitate the patient in all phases of recovery.

To date, no studies in the literature have verified the use of Gloreha Sinfonia in patients with SCI: however, studies using this device have found improvements in reducing spasticity, pain, and subject-reported symptoms of heaviness and stiffness, recovery of fine manual dexterity and strength, and reduction of arm disability in subjects with poststroke hemiparesis [14,15,16].

This case report aims to describe the potential of robotic hand treatment through the use of the Gloreha Sinfonia exoskeletal glove in association with conventional physiotherapy treatment in a patient suffering from traumatic tetraparesis.

## 2. Materials and Methods

The study presented is the first in the literature to report an experience related to the use of a robotic exoskeletal device for the hand on a patient suffering from chronic cervical SCI. The reported experience concerns a young patient, aged 23, who reported the SCI at the age of 14.

The present work is written following the CARE Guideline for Case Report [17].

### 2.1. Patient Information

The patient (F. L.) is a 23-year-old man who presents an incomplete spinal cord injury (ASIA B) level C6, resulting in tetraparesis.

The trauma to the spine occurred in 2014 (patient’s age: 14) during a dive into the sea with an impact on the sand, which was followed by a loss of consciousness of unspecified duration. After the trauma, the patient was taken by air ambulance to the IRCCS Agostino Gemelli University Polyclinic Foundation, IRCCS of Rome, where an MRI was performed, which highlighted the presence of a C5 fracture and a C6 compound somatic fracture, with C4–C6 medullary edema. For this reason, on 5 July 2014, an anterior cervical arthrodesis was performed.

During an initial physiatric and physiotherapy evaluation, the patient presented hypotonia of the lower limbs, with patellar and achilles areflexia. Tactile and pain sensitivity, as well as proprioceptive sensitivity, were preserved. As regarding upper limbs, osteotendon reflexes were valid and symmetrical, and there was not tone deficit. The patient also presented a complete strength deficit (Medical Research Council—MRC 0/5) of bilateral flexor-extensor muscles of the fingers, interosseous muscles, and lower limb muscles; a grade 3/5 of MRC was given for flexor and extensor muscles of the carpi, biceps, and bilateral triceps, and a score of 4/5 was given for the bilateral trapezius’ evaluation.

On 21 July 2014, following the clinical stabilization, the patient was discharged from the Orthopedics and Traumatology department of the Agostino Gemelli hospital and admitted to the Spinal Unit of the Montecatone hospital, where he was discharged on 23 December 2014, with five months of total time of hospitalization.

At the end of the hospitalization in the Spinal Unit, the patient presented:

- Normal range of motion (ROM)
- Sufficient static and dynamic trunk control
- A slight increase in tone of the lower limbs, in particular of the knee flexors and extensors, hip adductors, and bilateral tibiotarsal plantiflexors
- Residual motor skills absent in the lower limbs, left triceps, wrist flexors, and hand muscles
- Presence of proximal motor quotas at the level of the pronators and wrist extensors (MRC 4-5/5) and a hint of contraction of the right triceps (MRC: 1/5).

Furthermore, he required minimal assistance in transfers (supine-lateral decubitus and bed to wheelchair) and in dressing and was fed independently with the use of adapted cutlery. He used the wheelchair independently on flat (indoor and outdoor) or slightly sloping terrain, managing to overcome very low sidewalks autonomously.

From 11 February 2015 to 11 June 2015, he was treated at the Santa Lucia Foundation as an outpatient, where he underwent four motor rehabilitation sessions a week, two of which were in water. At the final evaluation, the patient showed an improvement in trunk control, autonomy in transitions from supine to sitting, transfer to wheelchair, and use of the self-propelled wheelchair. In addition, lower limb motility was absent with the presence of spasticity, as well as with the presence of clonus. In the upper limbs, there was spontaneous movement bilaterally, in particular: deltoid force (F): 4, biceps F: 5, triceps F: 3, extensor carpi F: 4, flexor carpi F: 1, flexor digitorum F: 2.

Subsequently he continued the hydrokinesis therapy for another year, associating it with home physiotherapy five times a week.

Since 2018, he has been carrying out rehabilitation therapy of the upper limb with Gyrotonic^®^ (International Headquarters, Dingmans Ferry, PA, USA), a tool that allows functional training, which stimulates the joints and exercises the muscles and allows the body to stretch and strengthen at the same time.

In 2021, the patient underwent a cycle of 33 session of gait rehabilitation with an Ekso NR exoskeleton (Ekso Bionics, San Rafael, CA, USA) at the Campus Bio-Medico University Hospital Foundation in Rome.

### 2.2. Clinical Findings

During the physical examination, conducted on 20 July 2022, the patient presented a good level of autonomy in the ADLs (SCIM total score: 63/100), despite the limitations found in the aspects of physical functioning, evaluated both with the SF-36 (5/100 of the Physical functioning sub item) and with the SCIM (mobility 16/20): the patient had good trunk control and a margin of strength in the upper limbs, such that it was possible to include him in the training with Gloreha. Furthermore, there was a tendon retraction at the level of the finger flexors, which led to joint limitation at the last degrees of extension. The patient had no hypertonia (Ashworth scale: 0/5), and he was autonomous in bed–wheelchair transfers (Trunk control test for SCI: 23/24) and in short and long-distance rides with a super light self-propelled wheelchair.

Despite the level of lesion, the patient had good functionality of the organic system, assessed with the CIRS, with a SI (Severity Index) equal to 0.9 and a CI (comorbidity Index) of 4.

Moreover, no problems related to emotional, social, and pain aspects were reported.

### 2.3. Intervention

The protocol provided a six-week treatment, consisting of two or three sessions per week for a total of 15 sessions. The rehabilitation sessions lasted one hour, divided equally between robotic rehabilitation (approximately 30 min, 15 min per side), in which the Gloreha Sinfonia exoskeletal glove (Idrogenet S.R.L., Brescia, Italy) was used, and conventional treatment (approximately 30 min, 15 min per side) aiming at restoring the correct muscle lengths, as well as strengthening and improving muscle recruitment.

#### 2.3.1. Robotic Therapy

**Figure 1:**
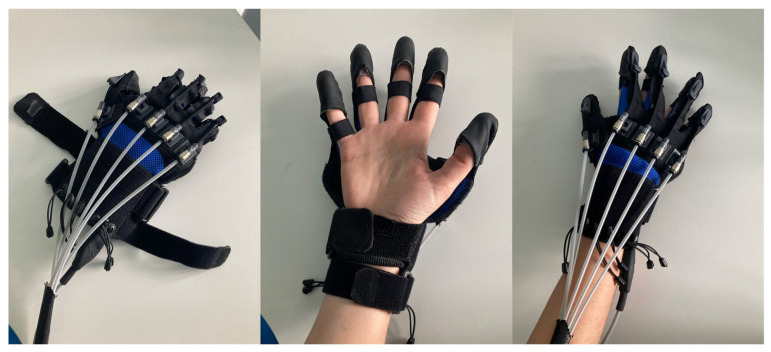
The Gloreha robotic glove.

The Gloreha Sinfonia (Idrogenet S.R.L., Brescia, Italy) is a robotic device for the neuromotor rehabilitation of the upper limb, which can facilitate the patient in all phases of recovery [18] (Figure 1).

It can support the movement of the finger joints in passive, active-assisted, and active modes. It consists of a complete set of gloves, braces, and accessories for finger mobilization, a dynamic support to compensate the weight of the arm, a stimulating software equipped with three-dimensional animation, a voice guide and audio-video effects, a touchscreen PC, and an ergonomic table for performing functional exercises to allow its use with a wheelchair.

The device allows the execution of the following exercises:

1. Passive mobilization exercises (the movements are carried out entirely by the device);
2. Active-assisted exercises with graphic interface (the patient trains in flexion extension of the fingers thanks to motivating games; the motors support and integrate the patient’s voluntary movements only to the extent necessary) or with real objects (the patient trains fine grip);
3. Interactive games (the patient can improve dexterity).

Moreover, the robot allows a constant measurement of motor performance and condition of the patient’s hand (active/passive ROM, movement speed, coordination and improvement in the execution of the various tasks). The robotic glove records all data associated with each patient and allows the operator to monitor the performance of each subject treated. Graphs show the trend of the obtained results exercise by exercise and session by session so that the patient can have immediate feedback on the progress achieved.

In this study, the robotic treatment with Gloreha was carried out in active-assisted mode, so the robot was calibrated and programmed to intervene in the performance of the gesture when the patient was unable to complete it independently. The exercises included in the protocol were selected on the basis of the patient’s residual functionalities and with the aim of improving his autonomy in ADL.

Table 1 shows the exercises proposed in the order of execution: the robotic treatment lasted 30 min, divided equally between the two sides.

**Figure 2:**
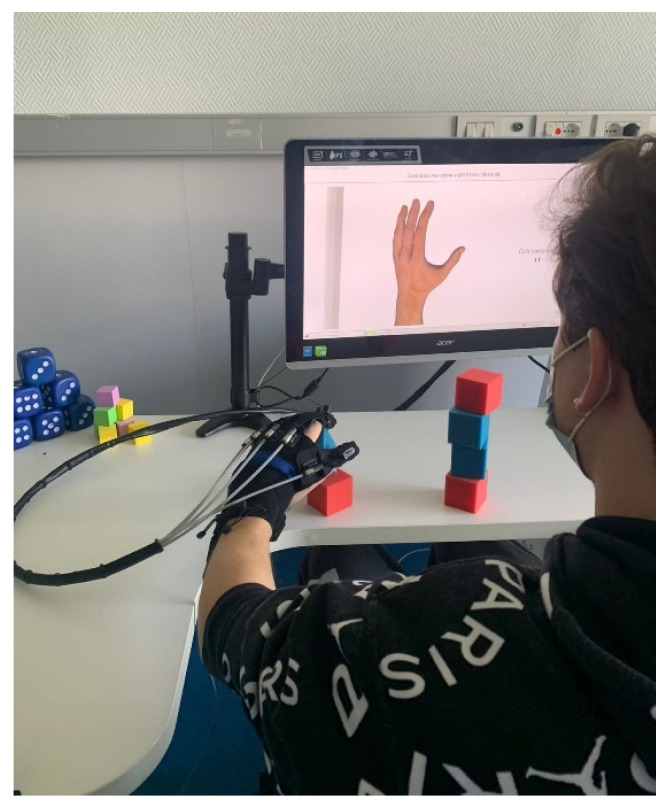
Build a column exercise.

It is important to highlight that the build-a-pyramid exercise was used only in the first two weeks of therapy. To increase the difficulty of the therapy, it was replaced by the build-a-column exercise for the next four weeks (Figure 2).

#### 2.3.2. Conservative Treatment

A conventional physiotherapy module was provided following the robotic treatment. The aim was focused on the maintenance of muscle lengths, on the muscle strengthening of the wrist and hand areas, on muscle recruitment, and on joint stability.

The exercises proposed in order of execution were:

1. *Muscle stretching of the tendons of the finger flexors and wrist flexors.* This exercise was proposed with the aim of decreasing the tendon retractions, which caused a limitation of the movement in extension to the last degrees of the 4th and 5th fingers.
2. *Muscle strengthening exercises in the flexion movement of the fingers and grip* (flexion and hold for 5 s, 10 repetitions for at least 2 sets). To improve the strength and resistance of the fingers, an instrument was used that was able to provide each finger with resistance in the flexion movement (closing of the hand) at the same time, through five independent springs. In this way, the activation was sought not only of the extrinsic muscles of the hand, but also of the intrinsic ones.
3. *Muscle strengthening exercises in the wrist extension movement* (15 repetitions for at least two sets). This exercise aimed at strengthening pure extension, trying to avoid compensatory movements—such as radial deviation, forearm supination, or shoulder flexion and abduction. It was performed against a resistance given by an elastic band, which limited its radial deviation, leaving the extension movement free.
4. *Rhythmic stabilization exercise of the wrist* (20 s for at least two series). In this exercise, the patient was asked to maintain a neutral wrist position while holding an object. The therapist created destabilizations through small pushes in all directions, which the patient had to counteract.
5. *Facilitation of muscle recruitment of the wrist flexors* (at least 10 repetitions). The therapist provided the patient with sensory-motor facilitation stimuli, with the aim of improving the activation and muscle recruitment of the forearm and wrist muscles in the flexion movement.

The treatment protocol was carried out on both the left and right limbs (15 min for each side), with a total duration of about 30 min.

### 2.4. Outcome Measurement

The evaluation of the patient was carried out before the start of the treatment (T0—27 July 2022), at the end of the treatment to verify improvements compared to the initial condition (T1—27 October 2022), and two months after the end of the treatment (T2—27 December 2022) to verify the maintenance of the results achieved at T1.

**Figure 3:**
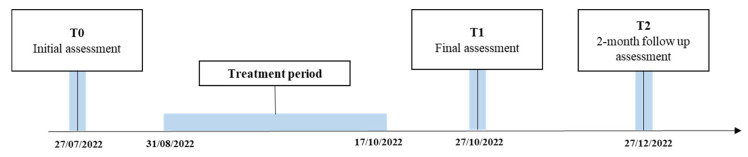
Timeline of the study.

Figure 3 reports the timeline of the study.

The clinical scales and evaluations used to verify the expected outcomes were the following:

- *Fugl Meyer Assessment for Upper Limb (FMA/UE)*. This scale is one of the core measures to be used in the evaluation of patients with central nervous system (CNS) pathologies, such as stroke and SCI, as it evaluates the residual functionality of the affected limb(s). A higher score represents a greater functionality of the limb. The scale is comprised of five domains with 155 items in total:
  - Motor functioning (0–66 points)
  - Sensory functioning (0–12 points)—Evaluates light touch on two surfaces of the arm and position sense for shoulder, elbow, wrist and thumb joints;
  - Joint range of motion of shoulder, elbow, forearm, wrist, and fingers (0–24 points);
  - Joint pain (0–24).
- *Disability of the Arm, Shoulder and Hand (DASH)*. This is a self-administered questionnaire that investigates the disability caused by the patient’s upper limb disorders in carrying out the ADLs. It consists of a total of 38 questions, divided into a main module (30 questions), which investigates hand functionality in ADLs and in recreational activities, the patient’s perception of disability in work and social activities, the presence of symptoms and difficulties related to limb and two optional modules, one question concerning working (four questions), and one question concerning sports/recreational activities (four questions). The score is expressed as a percentage from 0 to 100, where increasing the percentage will correspond to a greater disability.
- ***Nine Hole Peg Test (9HPT)***. This test is considered the gold standard for measuring manual dexterity in patients with upper limb disability. The test is performed by picking up the nine pegs, one at a time, with one hand, and inserting them into the nine holes provided. The test must be carried out as quickly as possible, since the measure used for the evaluation is precisely the time taken by the patient to complete the task.
- ***Range of Motion (ROM)***. Passive range of motion of the shoulder, elbow, wrist, and hand was measured using a goniometer. The measurements are expressed in degrees to evaluate all the movements of the upper limb joints.
- ***Medical Research Council (MRC)***. Upper limb muscle strength was measured with the MRC scale. In this assessment, the patient performs a movement, and the therapist offers an opposing resistance to it. A score is given from 0 (no contraction) to 5 (normal strength, movement against maximum resistance).

### 2.5. Force Assessment System Based on Virtual Reality Games

As an additional parameter to evaluate grip strength, a virtual reality (VR) game-based evaluation system was used to measure the patient’s improvements in force control and modulation [19,20]. This system involves the use of three piezoresistive sensors (FSR^®^ by Interlink Electronics, Camarillo, CA, USA), applied at the level of the distal phalanges of the I, II, and III finger, to measure the grip force exerted by each finger. The sensors are fixed on the fingertips by means of Velcro straps. In addition, a PLA plate printed in three dimensions (1 × 10^−3^ mm of thickness, 2 × 10^−2^ mm of diameter) was positioned between the sensor and the Velcro strap to uniformly distribute the force and fix the sensor to the finger. Sensors were calibrated using the Instron^®^ testing machine to relate force and output voltage, acquired through a custom-made printed circuit board.

The VR game, developed with Unity, represents an intuitive, clear, and engaging visual feedback for the user. Providing patients with biofeedback, in fact, allows improving the outcome of the treatment and promoting neuroplasticity [21].

In particular, the VR game comes in the form of a tracking task of three different waveforms (i.e., a “Ramp”, a “Square Wave”, and a “Sinusoid”) (Figure 2). The “Ramp” and “Square Wave” are composed of 10 discrete force levels to be reached (for the “Ramp”) and held (for the “Square Wave”) in a controlled way, uniformly distributed between the maximum and minimum force that could be applied, whereas the “Sinusoid” amplitude corresponds to the range between the minimum and maximum force.

The avatar of the game (i.e., a turtle) is controlled through the average force exerted by the three fingers on the object grasped by the participant (i.e., a wood parallelepiped 1.35 × 10^−1^ m × 6.5 × 10^−2^ m × 4 × 10^−2^ m).

Before starting the evaluation sessions, the maximum and minimum (i.e., the force corresponding to the touch of the object, without squeezing it) forces the subject can exert on the object must be recorded. In this phase, the patient was asked to apply his maximum grip force three times. The maximum force value to be reached during the exercises was set to 80% of the average maximum force value computed for the three fingers during the three repetitions.

**Figure 4:**
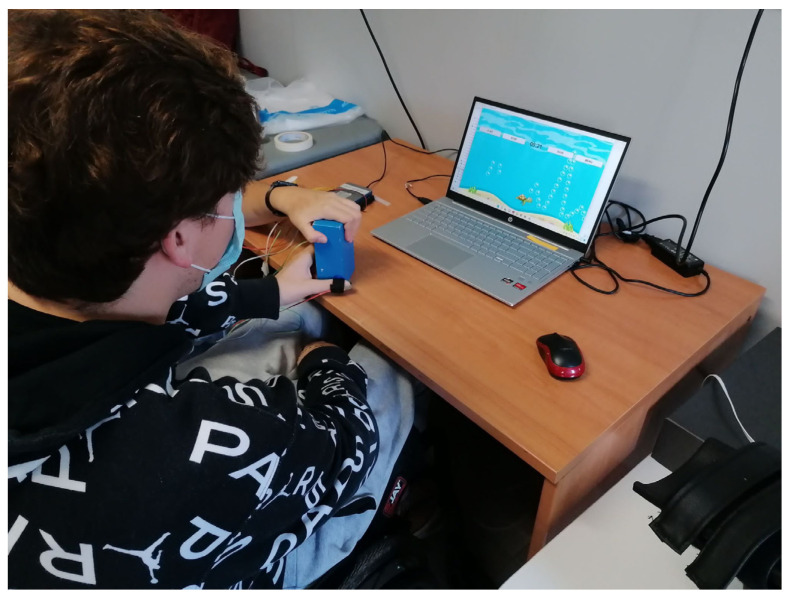
VR evaluation.

In the proposed VR games, the patient was asked to move the avatar vertically according to the exerted force in order to follow the target force pattern and collect all the objects (i.e., the bubbles) in the scene (Figure 4).

During the evaluation sessions, the patient performed two repetitions of each waveform in the first two sessions (i.e., T0 and T1) and three repetitions of each waveform in the last session (i.e., T2), with the lowest level of difficulty. Each repetition of the tracking tasks lasted 1 min. The duration was chosen in order to present to the user all the 10 different force levels to be reached in the “Ramp” and “Square Wave” exercises. Both the right and the left hand were assessed.

The normalized root mean square error (RMSE) was calculated to quantify the improvements in grip force control and modulation capability. It measures the error between the target and the exerted force patterns expressed as a percentage of the maximum force exerted by the patient in each evaluation session. In addition, the Kruskal-Wallis test [22] was applied to evaluate whether the differences in the RMSEs were statistically different between the three evaluation sessions.

## 3. Results

Results are reported in Table 2. As regards the functionality of the upper limb, no significant differences were reported between T0–T1 and T1–T2, as demonstrated by DASH assessment.

The motor evaluation of FM-UE also showed a slight improvement, passing from 58 to 59 for both limbs. This result was maintained at T2 for the left hand, while the right hand improved by an additional point (60 total) in the functional motor evaluation.

Manual dexterity was assessed using the 9HPT, in which the time taken by the patient to place the 9 cylinders in the nine holes was recorded.

An improvement of 3 s for the right hand and 8 s for the left hand was found at T1, while, at the evaluation at T2, the result worsened for the left hand (T0–T2 variation: +4.5 s; T1–T2 variation: +12.5 s) and improved further for the right one (T0–T2 variation: −21.6 s; T1–T2 variation: −18.6 s).

No changes were found in the sensitivity and pain assessments carried out using FM-UE (sensitivity intact and pain absent).

To verify variations at the joint level of the upper extremities, the ROMs of the shoulder, elbow, wrist, and hand of both sides were measured (Table 3). At the T1 evaluation, the only variation that was found was at the level of the extension of the proximal interphalangeal joints (IFP). This was impaired by about 10° bilaterally at T0, while an extension of 0° was recovered at T1. At the follow-up, the patient presented a bilateral reduction of shoulder abduction (135°). The other parameters remained stable, except for IFP extension, which changed from 0 to 20°, bilaterally.

As regarding muscle strength, no variations in MRC evaluation emerged between the two limbs, nor variations between the measurements at T0 and at T1, except for wrist extension and flexion movements of metacarpal phalangeal joint (MCF). As reported in Table 4, the previous results were maintained also at the follow-up evaluation. Therefore, a reduction in right elbow extension force was recorded at T2 assessment.

### Force Assessment

As regarding the force assessment system based on VR games, the maximum forces exerted by the patient in the three sessions are reported in Table 5. These values represent the average of the maximum forces recorded on the I, II, and III fingers. The maximum forces to be reached during the exercises were set to 80% of these values. As evident, the maximum forces increased from T0 to T2 for both hands.

**Figure 5:**
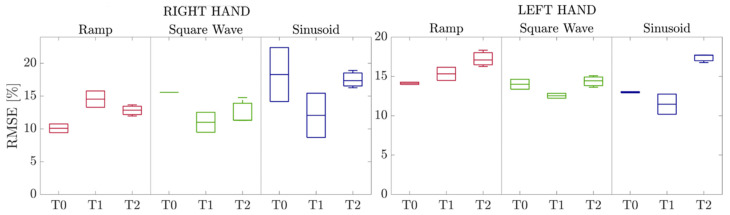
Boxplots of the RMSE in each session for both hands.

Figure 5 shows the boxplots of the RMSE in each session for both hands. Errors decreased for the “Square Wave” and “Sinusoid” between sessions T0 and T1. However, RMSEs tended to increase again between T1 and T2. Nevertheless, these differences were not statistically significant for the right hand during the three exercises in the three evaluation sessions T0, T1, and T2 (*p* = 0.63). For the left hand, instead, the Kruskal-Wallis test and multiple comparison analysis with Bonferroni correction [23] revealed that errors executed at session T2 were statistically higher than the errors at session T1 (*p* = 0.0079). Overall, median RMSEs calculated on the entire evaluation session (i.e., the three waveforms together) were 14.1%, 12.9%, and 13.6% for T0, T1, and T2 for the right hand, as well as 13.7%, 12.8%, and 16.7% for T0, T1, and T2 for the left hand.

## 4. Discussion

The aim of this study was to verify whether a six-week treatment protocol, carried out with robotic rehabilitation and physiotherapy, could bring appreciable benefits and improvements in the handling, reaching, and using of objects with regard to the strength and general condition of the hands in a patient with traumatic tetraparesis.

In the literature, it has been shown how the robotic approach in the rehabilitation of the hand of SCI patients can bring to a greater functionality related to grip force and manipulation of objects [24,25]. This is because, during the use of the exoskeletal robot, the movements were proposed in a repetitive, intensive, and targeted manner, thus stimulating more neuronal plasticity [10,11]

The evaluations carried out in this study showed a general improvement in the condition and use of the upper limb following treatment, which were also maintained at the follow-up (T2). In particular, the FMA-UE reported a slight improvement in functionality and passive movement for both the right and left limb with maintenance of intact sensitivity and absent pain.

This result is in line with the literature. In fact, Morone et al. [5] highlighted that robot-assisted rehabilitation might be considered promising training to improve muscle function in SCI.

Although no substantial differences were found in the DASH scale assessment, the patient reported an increase in the ability to handle and hold objects with less elbow and shoulder compensation, finding a greater use of the wrist and hand districts, especially the right side, which is the dominant side.

This result is confirmed by the dexterity test: in the evaluation carried out with the 9HPT, a reduction in the time of carrying out tasks was obtained in both hands at T1, while at T2 only, the right hand had further reduced the time of achievement of the test. The left hand, instead, returned to the starting condition.

This may be related to increased use of the right hand, i.e., the dominant one, in daily life activities. In fact, the use that the patient makes of the dominant side is greater. Studies on the role analysis of dominant and non-dominant hand in daily life have shown that the usage of the non-dominant hand is auxiliary, simpler, and greatly different from that of the dominant hand in healthy subjects [26].

It is possible to assume that the results obtained at the final evaluation were maintained at the follow-up for greater daily use of the right hand in more complex movements, similar to those carried out with the robot.

In fact, the assisted mobilization provided by Gloreha allowed for wider hand movements. This, in combination with muscle lengthening, this resulted in the achievement of less stiffness and joint limitation of the wrist and especially the 4th and 5th fingers of the hand, in which there was a decrease in tendon retraction and greater extensibility, also maintained at the follow-up evaluation.

ROM and force measurements (using MRC) at shoulder and elbow level showed a substantial stability, as these districts were not trained during treatment. Instead, slight changes were observed according to the MRC scale in the flexion-extension of the MCF joints, trained both with conventional treatment and by the assisted mobilization of the robot.

The force control and modulation capability of the hand were investigated by recording the grip force with piezoresistive sensors. The data collected indicated an increase in the maximum exertable grip force that doubled in the right hand and increased by 1.6 times in the left hand at T1. This improvement is also present at the follow-up evaluation, as the maximum force increased by 0.3 N for the right hand and 0.2 N for the left hand.

From the calculation of the errors committed in the force tracking of the trajectories in the aforementioned evaluation, data on the patient’s progress could be obtained. For the “Square Wave” and “Sinusoid”, errors decreased between T0 and T1 sessions, whereas RMSEs increased again between T1 and T2 for both hands (Figure 4). It is possible to assume that the increase in RMSEs at T2 may be related to the fact that the follow-up assessment was conducted two months after the conclusion of therapy with Gloreha Sinfonia, and the benefits brought by the treatment started to diminish.

Despite the increase in RMSEs, it is important to point out that the errors executed at session T2 were statistically higher than the errors in session T1 (*p* = 0.0079) only for the left hand. This could be related to the fact that the right arm is the dominant one: as the patient reported, his use of the right hand was higher than the left one. The daily use of the right hand may have helped preserving the improvements measured at T1, i.e., after the therapy.

Even though the difference in the magnitude of the errors was not statistically significant, the RMSEs measured on the entire evaluation session at T1 (12.9% for the right hand and 12.8% for the left hand) were one percentage point smaller than the RMSEs measured at T0 (14.1% for the right hand and 13.7% for the left hand), thus demonstrating that the combined robotic and traditional therapy contributed to increase grip force control and modulation capability. The increase in the measured RMSEs at T2 with respect to T1 was statistically significant only for the left hand, which is the non-dominant hand that is less used in daily life activities.

As described in the literature [27], these data, associated with those related to the increase in strength, allow us to deduce that the treatment carried out brought benefits in terms of grip force of the right hand, with consequent improvement in performing ADL.

Of course, the result obtained cannot be generalized. Recent studies evidence how rehabilitation strategies in individuals with incomplete tetraplegia, who have residual motor function below injury level, should focus on activity-based approaches to improve skilled movement performance [25].

This could be achieved by using robots such as Gloreha: thanks to its functionality and degrees of assistance, it can support the patient by helping him to develop residual functional skills, promoting patient autonomy and reducing the care load of the caregiver.

It would be interesting to deepen the use of Gloreha in association with traditional rehabilitation in patients with spinal cord injury with impaired upper limbs in order to confirm or not confirm the results obtained.

## 5. Conclusions

In conclusion, the treatment of the hand with the exoskeletal glove Gloreha Sinfonia, combined with a protocol of traditional physiotherapy, can have benefits in terms of functionality and dexterity in a patient with incomplete cervical SCI in the short term. Further studies involving larger samples are needed to be able to say that this approach can actually bring significant improvements to the clinical condition of the limb. Finally, it could be very useful to integrate the evaluation with surface EMG data to directly monitor muscle recovery.

## Figures and Tables

**Table 1:** Exercises with Gloreha.

Type of Exercise	Description	Aim of the Exercise
*Dice in the box*	To grab the dice and place them inside a box, and vice versa, take as well as to take them out of the box and place them on the table.	To seek the activation and recruitment of the whole limb, starting from the shoulder, elbow, and finally the hand.
*Build a pyramid*	To grab the dice and place them in order to build a pyramid on the table. Once the exercise was completed, the dice had to be returned to their starting point.	To improve the recruitment of the whole limb and the grip of the object.
*Build a column*	To grab the dice and place them in order to build a column on the table. Once the exercise was completed, the dice had to be returned to their starting point	To improve reaching, precision, and movement control, especially in the release phase.
*Pick the flower*	Take the flower on the screen, holding it and drop it into a box	To improve the functional grip of the 1st, 2nd, and 3rd finger. Therefore, to work on the fine grip of small objects, a small die was used (volume of 8 cm^3^).

**Table 2:** Results from assessment.

Assessment		T0	T1	T2
FM-UE				
RIGHT SIDE	Motor functioning Sensory functioning Joint ROM Joint Pain	58 12 23 24	59 12 24 24	60 12 24 24
LEFT SIDE	Motor functioning Sensory functioning Joint ROM Joint Pain	58 12 23 24	59 12 24 24	60 12 24 24
**NHPT**	Right Side Left Side	61 s 56 s	58 s 48 s	39.4 s 60.5 s
**DASH**		34%	33%	34%

FM-UE: Fugl-Meyer for upper extremity; NHPT: nine-hole peg test; s: seconds.

**Table 3:** ROM of Upper limbs.

District	ROM Left (°)			ROM Right (°)		
	T0	T1	T2	T0	T1	T2
**Shoulder**						
Flexion	180	180	180	180	180	180
Extension	60	60	60	60	60	60
Abduction	180	180	135	180	180	135
Horizontal adduction	45	45	45	45	45	45
Horizontal abduction	135	135	135	135	135	135
Internal rotation	70	70	70	70	70	70
External rotation	90	90	90	90	90	90
**Elbow-Forearm**						
Flexion	150	150	150	150	150	150
Extension	0	0	0	0	0	0
Supination	80	80	80	80	80	80
Pronation	80	80	80	80	80	80
**Wrist**						
Flexion	70	70	70	70	70	70
Extension	70	70	70	70	70	70
Ulnar Deviation	30	20	30	30	30	30
Radial Deviation	20	20	20	20	20	20
**Hand**						
MCP Flexion	90	90	90	90	90	90
MCP Extension	20	20	20	20	20	20
IFP Flexion	90	90	90	90	90	90
IFP Extension	10	0	20	10	0	20

IFP: proximal interphalangeal joint; MCP: metacarpal phalangeal joint.

**Table 4:** MRC of Upper limbs.

District	Left			Right		
	T0	T1	T2	T0	T1	T2
**Shoulder**						
Flexion	5	5	5	5	5	5
Extension	5	5	5	5	5	5
Abduction	5	5	5	5	5	5
Adduction	5	5	5	5	5	5
Horizontal adduction	5	5	5	5	5	5
Horizontal abduction	5	5	5	5	5	5
Internal rotation	5	5	5	5	5	5
External rotation	5	5	5	5	5	5
**Elbow-Forearm**						
Flexion	5	5	5	5	5	5
Extension	4	4	4	4	4	3
Supination	5	5	5	5	5	5
Pronation	5	5	5	5	5	5
**Wrist**						
Flexion	5	5	5	5	5	5
Extension	1	3	3	1	3	3
**Hand**						
MCP Flexion	2	3	3	2	3	3
MCP Extension	0	1	1	0	1	1
Finger abduction	0	0	0	0	0	0
IFP Flexion	1	1	1	1	1	1
IFP Extension	0	0	0	0	0	0
Thumb opposition	0	0	0	0	0	0
Thumb extension	0	0	0	0	0	0

IFP: proximal interphalangeal joint; MCP: metacarpal phalangeal joint.

**Table 5:** Maximum forces exerted by the patient in each evaluation session for the right and left hand.

Hand	T0	T1	T2
Right Hand	0.66 N	1.37 N	1.70 N
Left Hand	0.81 N	1.34 N	1.67 N

## Data Availability

No new data were created or analyzed in this study. Data sharing is not applicable to this article.
